# Highly sensitive detection of a *HER2* 12-base pair duplicated insertion mutation in lung cancer using the Eprobe-PCR method

**DOI:** 10.1371/journal.pone.0171225

**Published:** 2017-02-02

**Authors:** Yoshiaki Takase, Kengo Usui, Kimihiro Shimizu, Yasumasa Kimura, Tatsuo Ichihara, Takahiro Ohkawa, Jun Atsumi, Yasuaki Enokida, Seshiru Nakazawa, Kai Obayashi, Yoichi Ohtaki, Toshiteru Nagashima, Yasumasa Mitani, Izumi Takeyoshi

**Affiliations:** 1 Department of Thoracic and Visceral Organ Surgery, Gunma University Graduate School of Medicine, Maebashi, Gunma, Japan; 2 Division of Genomic Technologies, RIKEN Center of Life Science Technologies, Yokohama, Kanagawa, Japan; 3 K.K. DNAFORM, Yokohama, Kanagawa, Japan; CNR, ITALY

## Abstract

Somatic mutation in human epidermal growth factor receptor-related 2 gene (*HER2*) is one of the driver mutations in lung cancer. *HER2* mutations are found in about 2% of lung adenocarcinomas (ADCs). Previous reports have been based mainly on diagnostic screening by Sanger sequencing or next-generation sequencing (NGS); however, these methods are time-consuming and complicated. We developed a rapid, simple, sensitive mutation detection assay for detecting *HER2* 12 base pair-duplicated insertion mutation based on the Eprobe-mediated PCR method (Eprobe-PCR) and validated the sensitivity of this assay system for clinical diagnostics. We examined 635 tumor samples and analyzed *HER2* mutations using the Eprobe-PCR method, NGS, and Sanger sequencing. In a serial dilution study, the Eprobe-PCR was able to detect mutant plasmid DNA when its concentration was reduced to 0.1% by mixing with wild-type DNA. We also confirmed amplification of the mutated plasmid DNA with only 10 copies per reaction. In ADCs, Eprobe-PCR detected the *HER2* mutation in 2.02% (9/446), while Sanger sequencing detected it in 1.57% (7/446). Eprobe-PCR was able to detect the mutation in two samples that were undetectable by Sanger sequencing. All non-ADC samples were wild-type. There were no discrepancies between frozen and formalin-fixed paraffin-embedded tissues in the nine samples. *HER2* mutations detected by NGS data validated the high sensitivity of the method. Therefore, this new technique can lead to precise molecular-targeted therapies.

## Introduction

Lung cancer is one of the most common malignancies and causes of death worldwide[1]. Somatic driver mutations often confer a proliferative and survival advantage to cancer cells[2]. Somatic mutation in epidermal growth factor receptor (*EGFR*) is one of the driver mutations in lung cancer. Epidermal growth factor receptor tyrosine kinase inhibitor (EGFR-TKI) dramatically change the prognosis of patients with *EGFR* mutated adenocarcinoma. The median life expectancy for patients with advanced-stage non-small cell lung cancer (NSCLC) harboring an activating *EGFR* mutation increased to 20–30 months after EGFR-TKI therapy[3]. Another oncogene, erythroblastic leukemia viral oncogene homolog 2 (*ERBB2*), encodes human epidermal growth factor 2 (*HER2*) and is an ERBB receptor tyrosine kinase. *ERBB2* is a major proliferative driver that upregulates downstream signaling pathways. *S*omatic mutations of *HER2* are found in about 2% of lung adenocarcinomas (ADCs) [4–6]. The most common *HER2* mutation is a 12-bp duplicated insertion in exon 20 (80–100%), with ADCs showing a duplicated insertion of four amino acids (YVMA) at codon 775[4,7].

A previous study demonstrated that Trastuzumab (anti-HER2 drug) and Afatinib (an irreversible *ERBB* family blocker) had a disease control rate of 93% for trastuzumab-based therapy and 100% for afatinib in cases with *HER2* 12-bp duplicated insertion mutation[7]. The *HER2* mutation status has shown as a predictive and prognostic factor for lung cancer[8,9]. Therefore, analyzing the *HER2* mutation status could lead to the development of personalized therapies. While the importance of *HER2* 12-bp duplicated insertion mutation detection in lung cancer has been investigated previously [4,7,10–15], the efficiency of detection assays is still unknown.

Recently, we developed an original fluorescence real-time PCR procedure, referred to as the Eprobe-mediated PCR method (Eprobe-PCR)[16]. Eprobe is a hybridization-dependent fluorescence probe based on the quenching two-dye moieties under the condition of its single-stranded oligonucleotide and can be applied to sequential quantitative PCR and melting curve analysis in the same reaction tube with a real-time PCR machine[16,17]. This study focused on evaluating the feasibility of Eprobe-PCR for detecting *HER2* duplicated insertion mutation and attempts to compare the detection efficiency with Sanger sequencing and NGS methods.

## Materials and methods

### Reagents

DNA oligonucleotides, primers, and Eprobe, provided by K.K. DNAFORM (Yokohama, Japan), were stored as 100 mM stock solutions in TE buffer (10 mM Tris-HCl, 1 mM EDTA, pH 8.0). The Eprobe-PCR primers and probes constructed in this study are available as a “Primer & Eprobe set for *HER2* Exon20 YVMA insertion” via K.K. DNAFORM. Wild-type human genomic DNA was purchased from Promega (Tokyo, Japan). The target region of the *HER2* gene exon 20 insertion mutation was cloned into the plasmid pGEM-T (Eurofins Genomics, Tokyo, Japan and stored in TE Buffer at −20°C. Wild-type and mutant clone sequences were verified on a 3130xl Genetic Analyzer (Applied Biosystems, Foster City, CA, USA). EASY Dilution^®^ (Takara, Shiga, Japan) was used to prepare serially diluted plasmid DNAs.

### Experimental condition of Eprobe-PCR

**Fig 1:**
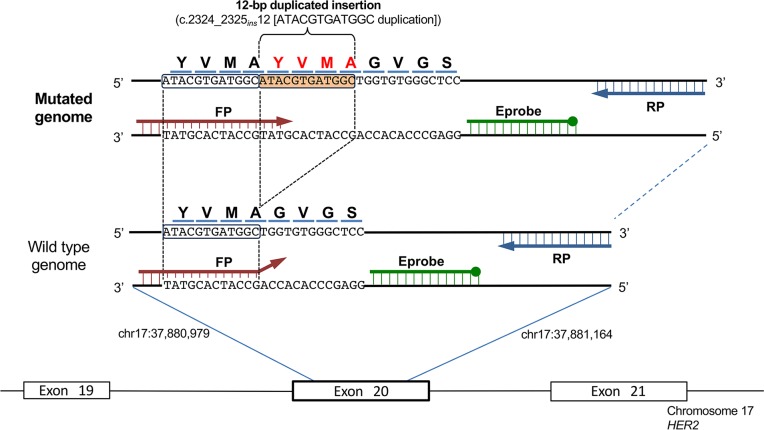
Primer sets and Eprobe design for *HER2* mutation detection. Schematic diagram of primers for the detection of the *HER2* 12-bp duplicated insertion by Eprobe-PCR. The orange box is the duplicated insertion. The forward primer for detection of the mutant-type allele contains the full sequence of *HER2* across the region known to be a frequent insertion site. The green bar is the Eprobe. The 3’ end-filled circle Eprobe shows the blocker that prevents primer extension during PCR.

The specific primers and Eprobe for the *HER2* 12-bp duplicated insertion are shown in Fig 1. The forward primer was designed to enrich the mutant-type using the principles of allele-specific PCR. A primer and probe design tool (Edesign[18]) was used to design all primers and probes.

A condition of asymmetric PCR (forward primer: reverse primer = 1:5) was used to detect amplification with Eprobe hybridization. The PCR products were a 100-bp amplicon, consisting of the *HER2* 12-bp duplicated insertion sequence. The forward primer was designed to be the mutation detection primer, with homology to the mutant nucleotide at the 3’ terminal position. The Eprobe-PCR reaction mixture was composed of the following reagents: 10 μL of 2×E-Taq PCR Mix (K.K. DNAFORM, Yokohama, Japan), 1.0 μL of diluted template DNA (10 ng/reaction), 0.1 μM of forward primer, 0.5 μM of reverse primer, and 0.4 μM Eprobe, in a total volume of 20 μL. The Eprobe-PCR temperature conditions were configured after heat-activation of the hot-start enzyme for 30 s at 94°C, followed by 50 cycles of three-step incubations at 94°C for 5 s, 64°C for 5 s, and 72°C for 7 s. Amplification signals were detected during the annealing step of each cycle at 64°C. The reactions were run with real-time PCR (LightCycler480, Roche Diagnostics, Mannheim, Germany) using a SYBR Green I filter (483 nm). After amplification, the crossing point (Cp) value of each amplification curve was calculated by the second derivative maximum method. We defined the threshold as Cp value <50.

### Clinical samples

Six hundred thirty-five tumor samples were obtained from patients with lung cancer resected at Gunma University Hospital (Gunma, Japan) between 2002 and 2014. The histological types included adenocarcinoma, squamous cell carcinoma (SCC), small cell lung cancer (SCLC), adenosquamous carcinoma (AS), and large cell neuroendocrine carcinoma (LCNEC). We defined all histology except for adenocarcinoma as ‘non-ADC’. All samples were immediately frozen after surgical resection and stored at −80°C until DNA extraction. Genomic DNA was extracted from a 3–5-mm cube of frozen sample using a DNeasy Blood & Tissue Kit (Qiagen, Valencia, CA, USA) according to the manufacturer's instructions. After extraction, the DNA was diluted in TE buffer to 50–100 ng/μL and stored at −20°C. For DNA extraction from formalin-fixed paraffin-embedded (FFPE) tissues, two or three pieces of 4-μm-thick sections were sliced from the block with rich tumor components. The tumor area of the section marked on the corresponding hematoxylin and eosin stained slide was macrodissected to reduce normal tissue contamination. Genomic DNA from the FFPE samples was extracted using a QIAamp DNA FFPE tissue Kit (Qiagen) according to the manufacturer's instructions. We added RNase during the DNA extraction. The isolated DNA concentration was adjusted to 10–20 ng/μL with TE buffer. This study was approved by the Institutional Review Board for clinical trials of Gunma University and human research ethical committee of RIKEN. Informed consent from all patients for clinical study of *HER2* genotyping were obtained in writing. Overall, this study was conducted according to the Declaration of Helsinki Principles.

### Sanger sequencing

PCR reactions for the target were performed in a final volume of 20 μL, containing 10 μL of AmpliTaq Gold 360 Master Mix (Applied Biosystems), 500 nM of each primer (forward primer: 5’-GTTTGGGGGTGTGTGGTCT-3’ and reverse primer: 5’-CCTAGCCCCTTGTGGACATA-3’), and 20 ng of isolated genomic DNA from the frozen tissue specimen. Thermal cycling conditions included preincubation at 95°C for 10 min, followed by 40 cycles at 95°C for 15 s, 60°C for 30 s, 72°C for 20 s, and an additional incubation at 72°C for 7 min. PCR products were purified using the QIAquick PCR Purification Kit (Qiagen) and processed for cycle sequencing with ABI PRISM BigDye Terminator (version 3.1, Applied Biosystems, CA, USA) and the same primers used in the first PCR. Sequence data were generated using the 3130xl Genetic Analyzer. All tumor samples were confirmed by direct sequencing of the PCR product for both DNA strands. We used “4Peaks” to view and edit the sequence trace files (http://nucleobytes.com/4peaks/).

### Next-generation sequencing

Amplicon sequences using the next-generation HiSeq2500 sequencer (Illumina, San Diego, CA, USA) were performed on *HER2*. Isolated genomic DNA samples from 635 lung cancer specimens were applied to the first PCR reaction with target-specific primers. To add the sequencing adaptor harboring dual-index sequences for indication of each individual specimen, a second PCR (overlapping PCR) was performed with the 10-fold-diluted first PCR product, the sequencing adopter, and the common adopter extension primers. The details of each primer and PCR conditions are shown in Supplementary Methods. The products of the second PCR were purified by Ampure™ XP (Agencourt, Beverly, MA, USA), according to the manufacturer's instructions. DNA concentrations of all purified second PCR products were investigated by Dropsense™ 96 UV/VIS droplet reader (Trinean, Gentbrugge, Belgium). Purified products from the 95 specimens and positive controls were mixed with equal concentrations, and 8 pM DNA was applied to the HiSeq 2500 sequencer (Illumina). Cluster generation was performed by cBot cluster generation system (Cluster Kit v3-cBot-HS, Illumina). One hundred base pair paired-end raw data were generated by the Illumina HiSeq 2500 sequencer with HiSeq Control Software (v2.0.5, HCS) and real-time analysis (RTA, v1.17.20). Raw data were de-multiplexed by Illumina bcl2fastq software (v1.8.4) using a dual-index sequence. Burrows–Wheeler aligner (BWA, v.7.10-r789)[19] was used to align each de-multiplexed data to reference sequences based on human genome Hg19. Aligned data were converted from the same format data into bam format data with Samtools software (v0.1.18)[20], and pileup data were generated from the bam format data. Varscan software (v2.3.7)[21] was used to identify pileup nucleotide counts at each aligned base position against the reference sequence. We counted the total reads and the 12-bp insertion reads for each specimen (shown in S2 Table). The mutation ratio was calculated as the percentage of the 12-bp mutation read counts in the total read counts. We defined the thresholds as mutation ratio>0.2%.

## Results

### Establishment of Eprobe-PCR for detection of the *HER2* 12-bp duplicated insertion

Because *HER2* 12-bp duplicate insertion is a somatic mutation, highly sensitive detection procedures are needed to detect the mutation in heterogenic tumor specimens. Mutant-specific primers were used as the basis for detection of *HER2* 12-bp duplicated insertion by Eprobe-PCR. The annealing region of forward primer is located from adjacent upstream sequence of the mutation site, which completely anneals to the variant of the 12-bp insertion (YVMA) only at 3’-end of the primer. Four bases of 3’-end of the primer have mis-match to wild type genome without insertion (Fig 1). The reverse primer and Eprobe were also optimized for use with the forward primer through “Edesign”[18], which is able to design adequate PCR primers to amplify the target sequence (100 bp) without false positive amplification. With an optimized primer set, an Eprobe was located in the target sequence with the same direction as the forward primer (Fig 1). To obtain effective fluorescent signal by Eprobe hybridization to the amplified product, asymmetric PCR conditions (forward primer: reverse primer = 1:5) were applied to ensure that the complementary DNA strand was actively amplified against the designed Eprobe.

**Fig 2:**
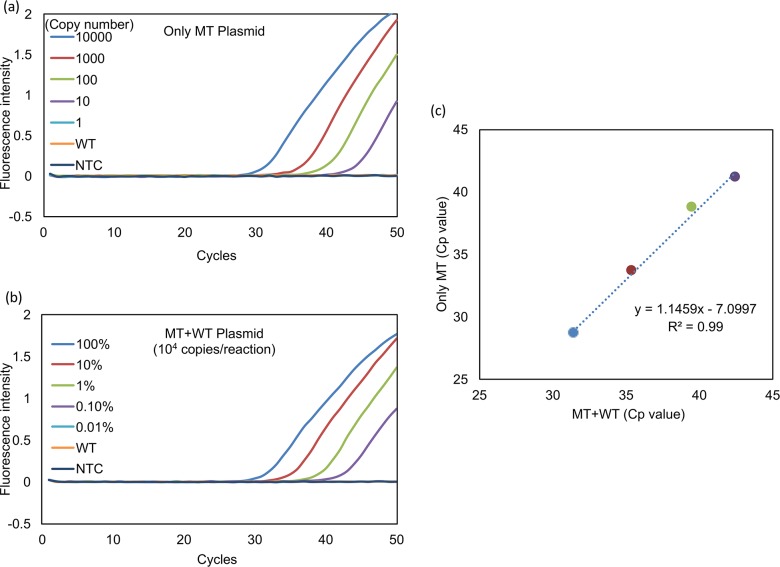
Sensitivity of Eprobe-PCR for detecting *HER2* 12-bp duplicated insertion. MT: *HER2* 12-bp duplicated insertion mutation type, WT: *HER2* wild type, NTC: No template control (diluted water). (a) Evaluation of mutated genome amplification. The blue line indicates MT only plasmid DNA at 10,000 copies per reaction, red: 1,000, green: 100, purple: 10, light blue: 1, orange: WT plasmid DNA, black: NTC. The light blue line shows no amplification. It overlaps with WT and NTC lines. (b) Sensitivity of 12-bp duplicated insertion detection in heterogenetic conditions. The blue line indicates MT only plasmid DNA at 10,000 copies per reaction, red: 1,000, green: 100, purple: 10, light blue: 1, orange: WT plasmid DNA, black: NTC (diluted water). The total copy number for each was adjusted to 10,000 copies per reaction. The light blue line shows no amplification. It overlaps WT and NTC lines. (c) Cp (crossing point) values of two experiments (a) and (b) were calculated by the second derivative maximum method in the LightCycler480. The data were then transferred to Microsoft Excel (Microsoft, Redmond, WA, USA) and Cp values were evaluated.

The quantitative sensitivity of the Eprobe-PCR for *HER2* mutation detection was evaluated *in vitro*. Serial copies of the *HER2* mutation plasmid DNA (MT plasmid DNA) containing 10000, 1000, 100, 10, and 1 copy per reaction and the template DNAs were applied to the Eprobe-PCR. The assay was able to detect 10 copies of MT plasmid DNA within 50 PCR cycles, as shown in Fig 2A. Next, we investigated the minimal detection limits of Eprobe-PCR from the aspect of mutation detection level from heterogenic sample. As template DNA to mimic tumor heterogeneity, we mixed 10000 copies of MT and WT plasmid DNAs to prepare 100, 10, 1.0 and 0.1% mutated template DNAs, respectively. Eprobe-PCR was able to detect only 0.1% in 50 cycles under the heterogenic condition (Fig 2B). Cp values were then compared between both template conditions (Fig 2C), and a strong correlation (R^2^ = 0.99) was indicated. These results suggest that the established primer set has high specificity and sensitivity for *HER2* 12-bp insertion mutation when at least 0.1% of the DNA contains the mutation. Fig 2A and 2B show that the primer set was incapable of amplifying the negative template control or wild-type DNA in 50 PCR cycles. Further examination revealed that even excessive amounts wild-type DNA (<100 ng/reaction) did not result in false-positive detection (data not shown). Therefore, the Eprobe-PCR method was easily able to discriminate *HER2* 12-bp insertion mutations from wild-type *HER2* with high accuracy.

### *HER2* mutation detection for lung cancer by Eprobe-PCR and comparison with NGS and Sanger sequence

Next, the established Eprobe-PCR *HER2* mutation analysis was used to identify somatic mutations in lung cancer samples. Human genomic DNA was isolated from frozen clinical specimens (635 lung cancer tumors). Eprobe-PCR analysis was performed using 10 ng of DNA, which corresponded to around 3000 copies of human genomic DNA. The Eprobe-PCR results (Table 1) revealed *HER2* 12-bp insertion mutations in 9 out of 635 lung cancer specimens (1.4%). Especially, all of the mutation positive specimens were categorized in ADC (n = 446; 9/446 or 2.02%, Table 2).

**Table 1:** Evaluation of the nine mutant tumors detected by Eprobe-PCR.

Sample name	Eprobe	Sanger	NGS		
			Read numbers		Mutation ratio (%)
			Total	Mutant	
Ad071	**MT** (32.5)	**MT**	111374	22994	20.65
Ad096	**MT** (38.0)	**MT**	92284	12852	13.93
Ad154	**MT** (45.0)*	**MT**	99378	17920	18.03
Ad238	**MT** (34.5)	WT	104054	8906	8.56
Ad253	**MT** (29.8)	**MT**	109436	81652	74.61
Ad264	**MT** (32.4)	**MT**	155348	87577	56.37
Ad341	**MT** (32.3)	WT	109595	34528	31.51
Ad367	**MT** (34.9)	**MT**	134855	11698	8.67
Ad385	**MT** (32.9)	**MT**	156677	30081	19.2

Mutation ratio = Mutant read number/Total read number×100. MT and WT show HER2 mutant-type (12-bp duplicated insertion) and HER2 wild-type, respectively. Numbers in parentheses indicate Cp values of Eprobe-PCR analyses.

* The genomic DNA concentration of Ad154 was quite low (0.2 ng/reaction) while the others were 10 ng/reaction.

**Table 2:** Comparison of three genotyping methods in all clinical samples.

Histological type	Method	Mutations /Total	%
ADC	Eprobe-PCR	9/446	2.02
	NGS	9/446	2.02
	Sanger	7/446	1.57
non-ADC	Eprobe-PCR	0/189	0
	NGS	0/189	0
	Sanger	0/189	0

To validate the above results, amplicon sequencing of all lung cancer specimens was performed using the Illumina HiSeq2500 sequencer. The *HER2* 12-bp insertion mutation ratio (*i*.*e*., sample heterogeneity) of each specimen was also investigated. An average of 58894 ± 45207 (mean ± sd) reads were obtained for each specimen. We set the cutoff value of 0.1% for the mutation ratio (S2 Table) to detect as mutation positive. All Eprobe-PCR results were supported by the amplicon sequencing results. The mutation ratios of the nine positive specimens ranged from 8.56 to 74.61% (Table 1). Thus, the validation suggested that our established Eprobe-PCR procedure had high accuracy and specificity for the *HER2* 12-bp insertion mutation detection in clinical genomic DNA samples.

**Fig 3:**
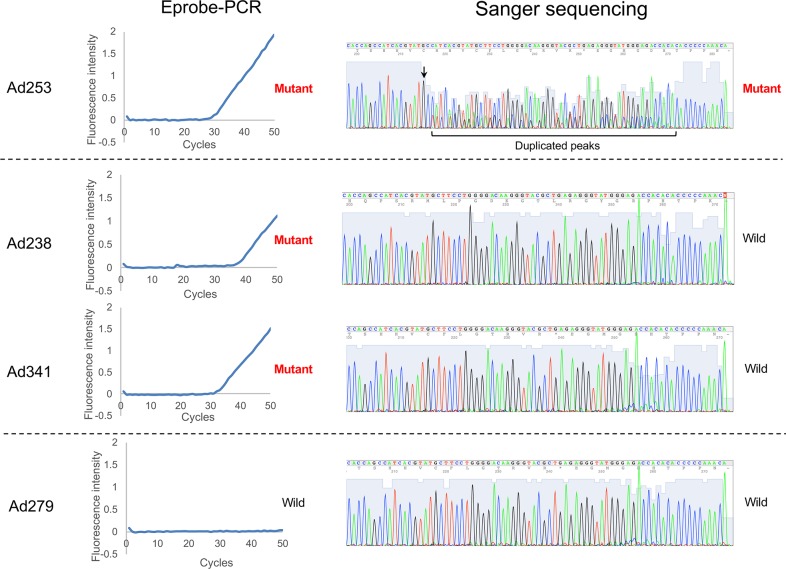
Comparison of Eprobe-PCR and Sanger methods. The left half of Fig 3 shows the amplification curves of Eprobe-PCR, and the right half shows the electrogram of Sanger sequencing. “4Peaks” was used to view and edit the sequence trace files (http://nucleobytes.com/4peaks/).

The Eprobe-PCR results were also compared to the conventional Sanger sequencing analyses. Seven specimens out of 635 showed *HER2* 12-bp insertion mutations by the Sanger sequencing (1.1%). All of the seven positive specimens were also determined to be positive by the Eprobe-PCR method (Table 2). Fig 3 shows both the raw Eprobe-PCR data and the Sanger sequencing for each specimen. Specimen Ad253 was a typical mutation identified by the both methods. The crossing point (Cp) value of the Eprobe-PCR for Ad253 was the lowest of all samples (Cp: 29.8). These results suggest that Ad253 had the highest percentage of mutated cells in the tumor sample, which correlated with the highest mutation ratio (74.61%) by the amplicon sequencing (Table 1). In the Sanger results for Ad253, dual electrogram peaks were found downstream of the mutated position (Fig 3). In contrast, Ad279 was determined as wild-type by the both methods; there was no amplification by the Eprobe-PCR and the Sanger electrogram indicated a single peak. In the results of specimens Ad238 and Ad341, the Eprobe-PCR detected the mutation in Ad238 (Cp: 34.5) and Ad341 (Cp: 32.2); however, we could not identify a dual peak with the Sanger method. The mutation ratio of Ad341 was 31.51%, but it was not the lowest ratio of the nine positive samples (Table 1).

### Mutation detection in FFPE samples

FFPE is a common method for storing tumor samples in clinical practice, and several testing methods, including histological and morphological evaluations, can be performed on FFPE tissue. Therefore, the applicability of genetic diagnostic procedures to FFPE samples is essential. We assayed the nine FFPE tumor samples which had been previously revealed to harbor *HER2* mutation by Eprobe-PCR in frozen specimens. *HER2* mutation were detected in the all nine samples (S1 Fig). In addition, wild-type genomic DNA samples extracted from FFPE tissues were not amplified by the assays (data not shown). Therefore, these results suggest that the Eprobe-PCR is applicable for *HER2* 12-bp insertion detection in both frozen and FFPE tissue samples. In this study, we confirmed that the Eprobe-PCR could detect *HER2* mutations in FFPE specimens.

### Patient characteristics according to *HER2* mutation status

The clinical characteristics of patients with *HER2* mutation are summarized in Table 3. Comparing the two groups (*HER2* mutant type vs. wild type). The *HER2* mutation group include younger patients (*P* < 0.0001), more never-smokers(*P* = 0.013) and smaller tumor in size (*P* = 0.015) than wild type group.

**Table 3:** Patient characteristics according to *HER2* mutation status.

		Total (n = 635)	*HER2* wild (n = 626)	*HER2* mutant (n = 9)	*p*-value*
**Age at diagnosis**					
	Median	70 (31–90)	70 (31–90)	58 (48–72)	
	<65	183	175	8	<0.0001
	≧65	452	451	1	
**Sex**					
	Male	380	377	3	0.1
	Female	255	249	6	
**Smoking status**					
	Never	228	221	7	<0.05
	Smoker	407	405	2	
**Tumor size (cm)**					
	Median	2.5 (0.4–14.0)	2.5 (0.4–14.0)	1.2 (0.7–4.5)	*<*0.05**
**Differentiation**					
	Well-moderate	481	472	9	0.233
	Poor-undifferentiated	113	113	0	
	Unknown	41	41	0	
**Histological diagnosis**					
	Adenocarcinoma (ADC)	446	437	9	0.42
	Non-ADC	189	189	0	
	Squamous cell carcinoma (SCC)	146	146	0	
	Adenosquamous carcinoma (AS)	3	3	0	
	Small cell lung cancer (SCLC)	23	23	0	
	Large cell neuroendocrine carcinoma (LCNEC)	16	16	0	
**p-Stage**^#^					
	0	2	2	0	0.49
	IA, IB	429	420	9	
	IIA, IIB	88	88	0	
	IIIA, IIIB	88	88	0	
	IV	12	12	0	
	Unknown	16	16	0	

^#^ Pathological stage according to the American Joint Committee on Cancer staging criteria.

* calculated by a chi-square test.

** calculated by a Mann–Whitney test.

## Discussion

In this study, the Eprobe-PCR was shown to be a highly sensitive procedure for detecting *HER2* 12-bp insertion mutation in lung cancer samples. For the genetic diagnosis on somatic mutations by PCR combined with fluorogenic probe, technologies such as TaqMan^®^ assays and Scorpion-ARMS^®^ are widely used worldwide. These methods can detect both point mutations and insertion/deletion mutations. These fluorogenic probes generally were designed for the specific sequence of mutation, however, the repetitive sequences (e.g. *HER2*) are often avoided to prevent miss-hybridization of the probes. The *HER2* 12-bp insertion mutation occurs a tandem repeat on *HER2*, therefore, design of fluorogenic probe which hybridizes this insertion sequence should not be selected in Eprobe-PCR. In this study, we designed the forward primer to match the insertion sequence at 3’-end and the reverse primer and Eprobe on non-variant region, optimizing the combination with the forward primer. This enabled detection of the *HER2* mutation without false negative or positive results. To validate the detection quality of the Eprobe-PCR method, amplicon sequencing analyses were performed by the next-generation sequencer. The results showed that Eprobe-PCR is a highly sensitive detection method that can detect 0.1% of the mutation in heterogenic lung cancer samples.

The Eprobe-PCR method was also tested on isolated DNA from FFPE-embedded samples. The results showed that this method can also be successfully applied to both FFPE and frozen tissue samples. FFPE storage often affects genetic diagnosis due to chemical modification by formalin or through DNA fragmentation[22]. To avoid these effects, the length of the targeted amplicon should be shortened. The fluorescent oligonucleotides using the Eprobe-PCR method have hybridization-mediated fluorescence and higher stability as double-stranded DNA after the hybridization (i.e. increase of Tm value) than normal oligonucleotide[16]. These Eprobe features allow for the design of primers with short amplicons. In addition, the length of the *HER2* 12-bp duplicated insertion in this study is less than 100 bp, which likely contributes to the success of this method in FFPE tissues. Therefore, the Eprobe-PCR method for detection of *HER2* 12-bp duplicated insertion mutations is a useful diagnostic tool in clinical practice.

To evaluate the effectiveness of the Eprobe-PCR, 635 lung cancer specimens were tested. The clinical characteristics of patients used in the study are summarized in Table 3, and all were evaluated for clinical significance with regard to the *HER2* duplication mutation. Previous reports have shown that the 12-bp duplicated insertion is the most common mutation (80–100%) in the all *HER2* mutation types[4,7]. *S*omatic mutations of *HER2* are found in about 2% of lung adenocarcinomas (ADCs) and is a driver mutation[4–6]. In our results, all *HER2* 12-bp duplicated insertions were categorized as ADC (2.0%, n = 446), and the mutations were not found in any other lung cancer type tested (SCC, AS, SCLC and LCNEC). This is consistent with the previous studies.

As other clinical characteristics, previous studies have shown an association of *HER2* mutations with never-smoker and femaleness[4,7,8,11]. In our study, the *HER2* mutant patients were comprised of significantly large number of younger people (<65 years) and fewer smokers compared with *HER2* wild-type patients. We found a higher tendency of the *HER2*-mutation in female patients (7 females in 9 mutation positive specimens, statistically not significant (*P* = 0.1)). These clinical characteristics in our study were similar to the previous studies. As an additional interesting feature, we found that *HER2* mutations significantly correlate with a small tumor size. Although tumor size depends on progression of the cancer at the time of sampling, *HER2* driver mutations may occur during early-stage of ADC lung cancers.

A previous study has reported that multiple types of somatic *HER2* mutations, including insertion/deletion and point mutations, were found in lung cancers[4]. In that study, the vast majority of the *HER2* mutations (92%, 24/26) were in-frame insertions in exon 20 that ranged from 3 to 12 bp, all nested in the most proximal region of the exon. Several studies have previously detected a *HER2* 3-bp insertion, but did not find this mutation to be clinically significant [5,11]. Currently, the Eprobe-PCR method described in this study only has specificity to the *HER2* 12-bp duplicated insertion and is not applicable for detecting other mutations. Previous studies in our lab showed that Eprobe-mediated melting curve analyses after PCR amplification provided different Tm values, referred from genotypes on the genome by locating the mutation site on Eprobe target sequences[16,17]. Thus, Eprobe-PCR technology is advantageous compared with other techniques, due to its ability to detect multiple mutation types. In addition, expanding the Eprobe-PCR to large-scale genetic analyses could reveal clinically significant genetic mutations that may lead to personalized cancer treatments.

NGS technology has had a large impact on research into cancer genomics due to its high throughput sequencing capacity. Whole-genome sequencing of various cancers using NGS technologies has led to the identification of numerous genetic alterations. The large capacity of NGS also provides highly sensitive detection of individual mutations, and NGS “cancer panels” have been developed to sequence the known cancer genes[20]. However, NGS cost can be prohibitive for cancer diagnosis. In addition, each biomarker requires a companion diagnostic for each anti-cancer drug, and the large amount of genetic information available can actually lead to confusion regarding appropriate treatment. Therefore, *in-vitro* diagnostics, such as Eprobe-PCR, should facilitate the detection of specific mutations.

In conclusion, we have developed a highly sensitive assay for detecting *HER2* 12-bp duplicated insertion mutations in lung cancer. This study clearly showed that this assay is simpler and more sensitive than the Sanger sequencing method. The Eprobe-PCR assay is a useful diagnostic application and can lead to additional targeted therapies for *HER2* 12-bp duplicated insertion mutations in lung cancer.

## Supporting information

S1 Fig Results of mutation detection by Eprobe-PCR with FFPE samples. (TIF)

S2 Fig Electrograms of Sanger sequencing. (PDF)

S1 File Supplementary materials. (DOCX)

S1 Table Primers list of the library preparation for amplicon sequencing. (XLSX)

S2 Table Amplicon sequencing results of HER2 12-bp insertion mutation by illumina Hiseq2500 sequencer. (XLSX)
