# Supplementary figures and images for: Highly sensitive detection of a HER2 12-base pair duplicated insertion mutation in lung cancer using the Eprobe-PCR method
Source: PLoS One. 2017 Feb 2;12(2):e0171225. doi: 10.1371/journal.pone.0171225 (PMC5289711; doi:10.1371/journal.pone.0171225)

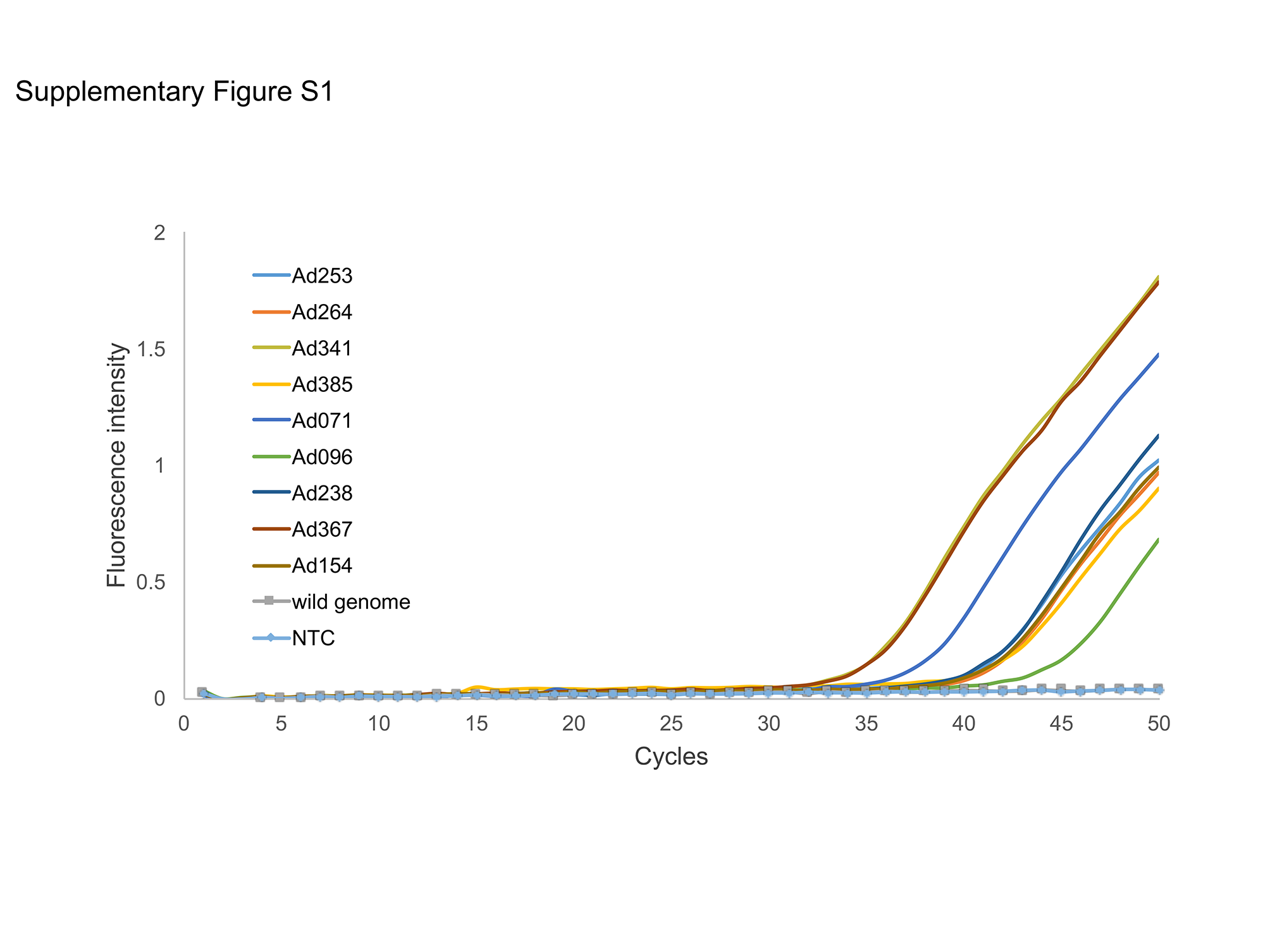

Supplement: S1 Fig — (TIF) [file pone.0171225.s001.tif]
