# Supplementary material for: Highly sensitive detection of a HER2 12-base pair duplicated insertion mutation in lung cancer using the Eprobe-PCR method
Source: PLoS One. 2017 Feb 2;12(2):e0171225. doi: 10.1371/journal.pone.0171225 (PMC5289711; doi:10.1371/journal.pone.0171225)
Supplement: S2 Fig — (PDF) [file pone.0171225.s002.pdf]

Supplementary Figure 2.

Ad071 (Mutant)

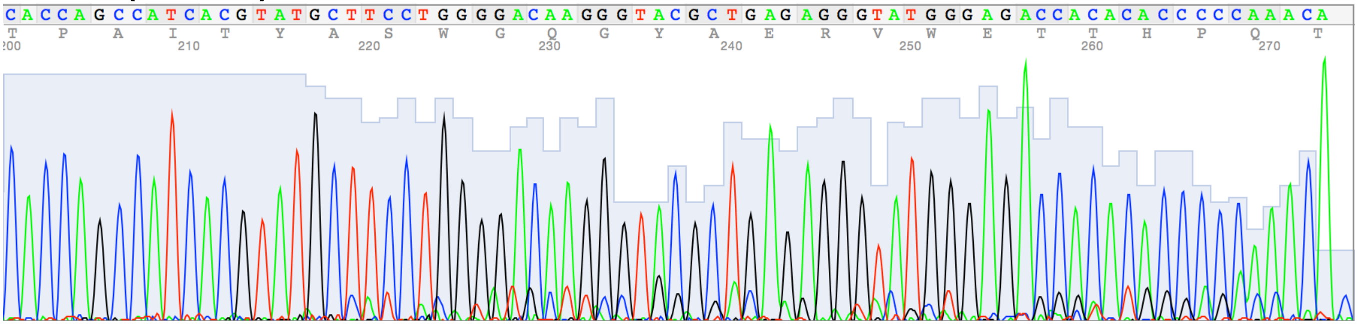

Ad096 (Mutant)

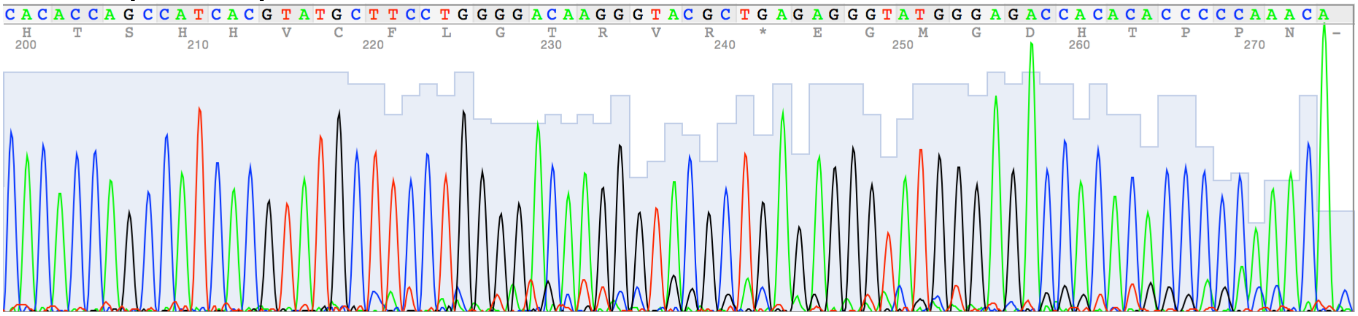

Ad154 (Mutant)

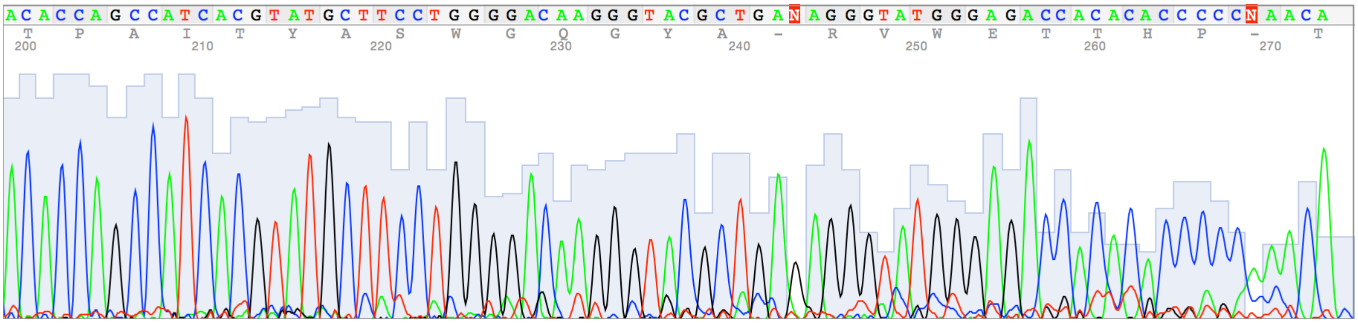

Ad238 (Wild)

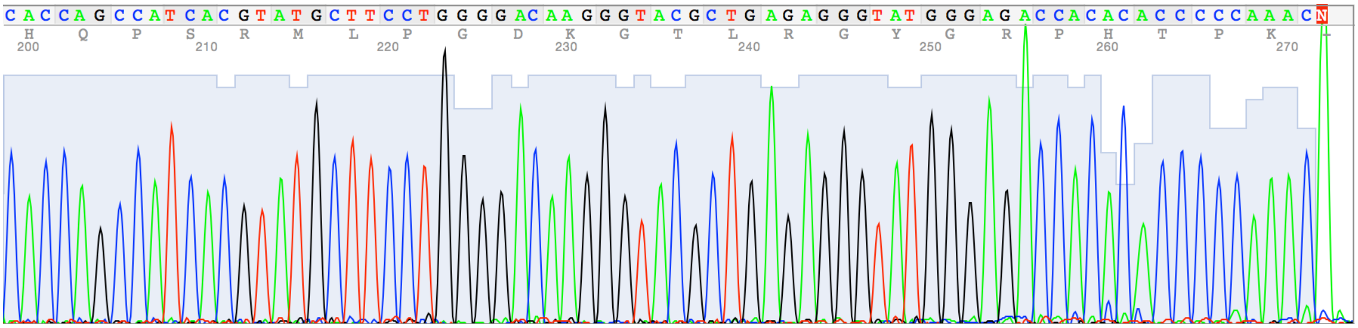

Ad253 (Mutant)

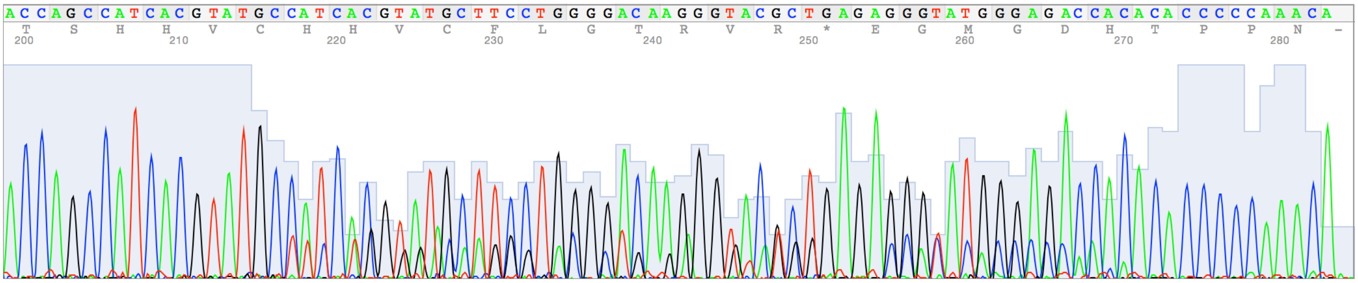

Continue to next page

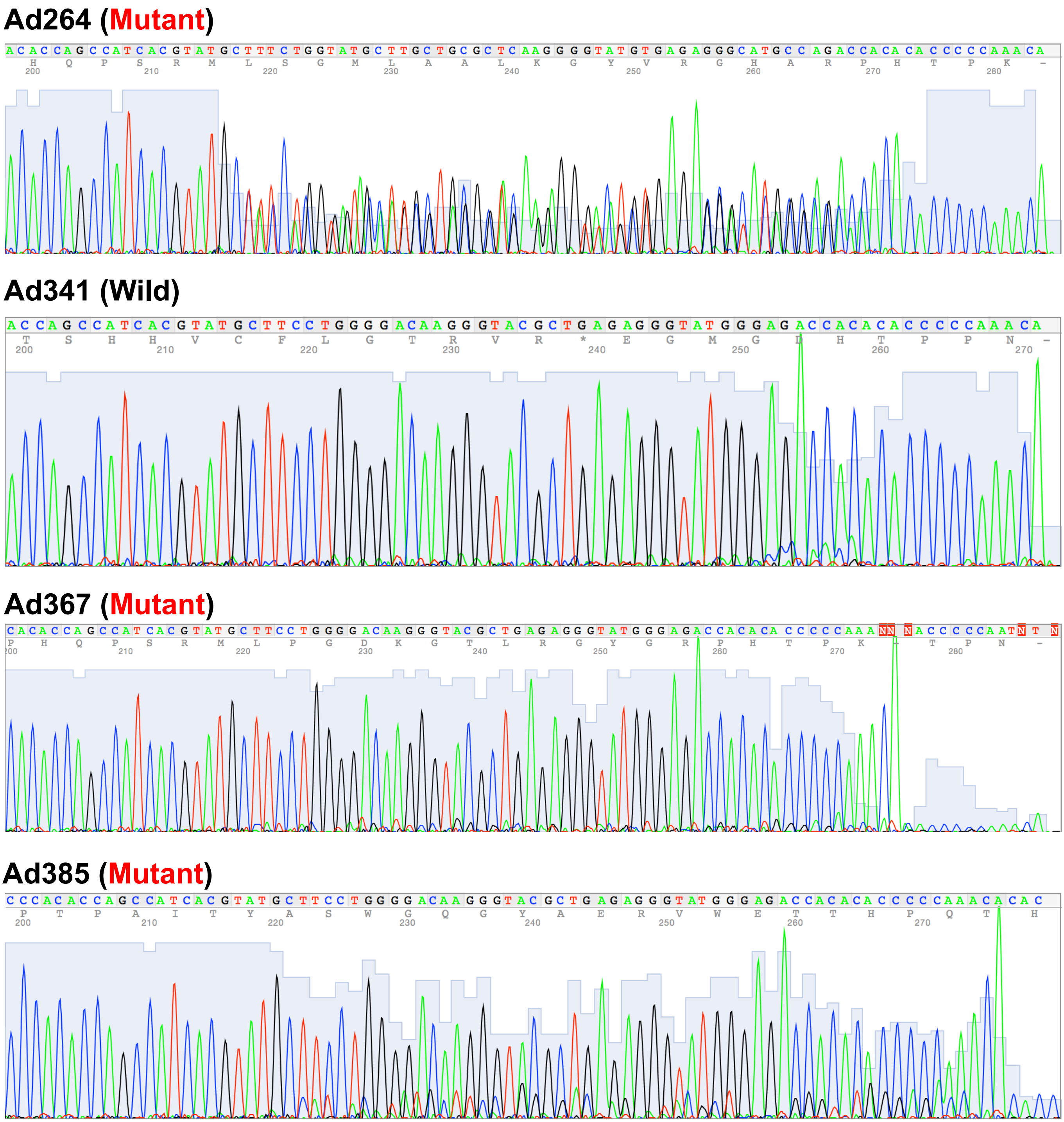

Supplementary Figure 2. Electrograms of Sanger sequencing.
The electrograms show the results for 9 mutation positive specimens determined by Eprobe-PCR method. The Sanger sequencing were carried out by the method described as "Materials and Methods" in the manuscript.
