# Supplementary material for: Highly sensitive detection of a HER2 12-base pair duplicated insertion mutation in lung cancer using the Eprobe-PCR method
Source: PLoS One. 2017 Feb 2;12(2):e0171225. doi: 10.1371/journal.pone.0171225 (PMC5289711; doi:10.1371/journal.pone.0171225)
Supplement: S1 File — (DOCX) [file pone.0171225.s003.docx]

Supplementary materials

**Supplementary Methods**:

PCR procedures for the library preparation of amplicon sequencing focused on *HER2*-duplicated insertion with illumina Hiseq2500.

**Supplementary Table S1** (an Excel file):

Primers list of the library preparation for amplicon sequencing.

**Supplementary Table S2** (an Excel file):

Results of the amplicon sequencing for *HER2*-duplicated insertion.

**Supplementary Figure S1** (an Powerpoint file):

Results of mutation detection by Eprobe-PCR with FFPE samples

**Supplementary Figure S2** (an PDF file):

Electrograms of Sanger sequencing.

***Supplementary Methods***

**PCR procedures for the library preparation of amplicon sequencing focused on *HER2*-duplicated insertion with illumina Hiseq2500.**

To prepare the library of amplicon sequencing, we used PCR approach divided two steps, target specific PCR around the position of *HER2*-duplicated insertion (1st PCR) and overlapping PCR for connecting the adapter sequences of illumina sequencing to the amplicon (2nd PCR). Basically, all PCR reactions were performed with PrimeSTAR^®^ HS DNA Polymerase (TAKARA BIO Inc., Shiga, Japan) and its attached reagents (5×PrimerSTAR buffer [Mg^2+^ plus] and dNTP solution).

(1) 1st PCR

In 1st PCR, we amplified the targeted DNA with the isolated genomic DNA from individual lung cancer specimen (frozen tumor) and *HER2* exon 20 specific primers (see Supplementary Table S1) by following PCR conditions.

As 1st PCR, we carried out 635 reactions for each lung cancer specimen with 96-well plate format (95 specimens and a negative control, total 7 plates). After the PCR, 5 µL of each PCR mixture was replaced to new 96-well plate, and diluted to 1/10 concentration with the distilled water for 2^nd^ PCR.

(2) 2^nd^ PCR

To add the illumina sequencing adapters to the 1st PCR product, we secondary performed overlapping PCR with 1st PCR products, adaptor oligos and common adaptor extension primers (see Supplementary Table S1). Each sequencing adaptor oligo includes 5’-common primer-annealing sequence, index sequence, and 3’-target specific sequence as following Figure #1.


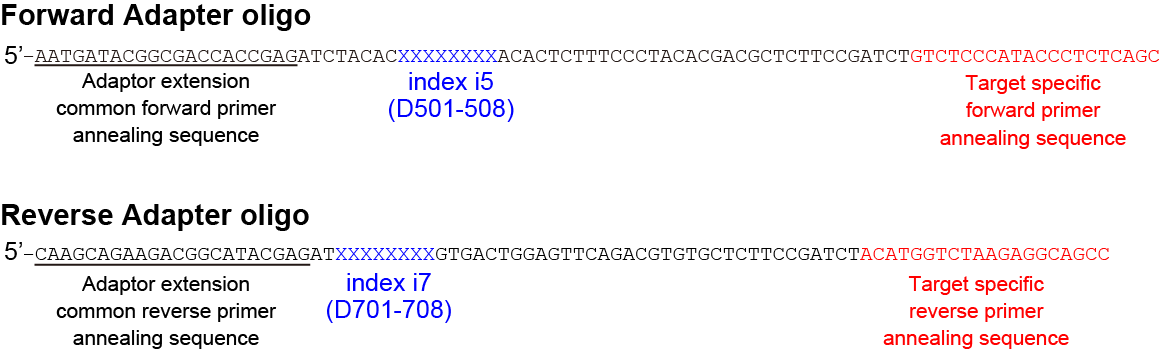


Figure #1. Schematic map of Adapter oligos

The sequences of these adapter oligos expect for the target specific sequences refer to the manufacture's instruction for illumina Truseq adapter. For the preparation of Adaptor oligo mixture consisted with a forward and a reverse oligos, we dispensed equal volume of 1 µM adoptor oligos to 96-well plate (final 0.5 µM oligos) by the following format based on series of index i5 and i7 (Figure #2).

Figure #2. Plate format of index i5 and i7

Sp#XXX indicates each specimen number, which corresponds with 1st PCR format in 96-well plate. NC is negative control.

After the preparation of adapter oligo mixture, we carried out 2nd PCR by following PCR conditions. As same as 1st PCR, total 635 reactions were carried out using seven 96-well plates.
